# Role of botulinum toxin-A in management of refractory idiopathic detrusor overactive bladder: Single center experience

**DOI:** 10.4103/0970-1591.40612

**Published:** 2008

**Authors:** N. K. Mohanty, Rajiba L. Nayak, Mohd. Alam, R. P. Arora

**Affiliations:** Department of Urology, VM Medical College and Safdarjang Hospital, New Delhi, India

**Keywords:** Anticholinergic, botulinum toxin, overactive bladder

## Abstract

**Background:**

Overactive bladder (OAB) is a bothersome condition affecting the quality of life, financial constraint on the individual, and community. Anticholinergic drugs cannot be used for long term due to adverse side effects. Botulinum toxin has recently shown promising and encouraging result in management of OAB.

**Aim:**

Aim was to study the safety, efficacy, tolerability, and duration of effect of 200 units of botulinum toxin in refractory idiopathic detrusor overactivity.

**Materials and Methods:**

Thirty-nine female patients (average age of 52 years) clinically and urodynamically diagnosed as idiopathic OAB were injected 200 units of botulinum toxin-A mixed with 20 ml of normal saline, intradetrusally at the rate of 1 mL at each site for 20 such sites sparing the trigone and ureteric orifices. Follow up at 3rd, 6th, 9th, and 12th month with clinical and urodynamical questionnaire was done.

**Results:**

There were 4 dropouts and 35 patients were evaluated, of which 30 patients (85.7%) showed improvement in clinical features like frequency, urgency, nocturia, and incontinence within 1 week of injection, which lasted for mean period of 7 months (varying from 6 to 9 months). Volume at first desire to void improved from median baseline of 104-204 ml and maximum cystometric capacity of bladder increased from mean baseline value of 205-330 ml. The detrusor pressure decreased by 49% from the baseline and postresidual urine volume increased by 30% of maximum cystometric capacity of bladder. There was no adverse effect on our patient.

**Conclusion:**

Intradetrusor injection of Botox-A in management of refractory overactive idiopathic bladder is not only safe and well tolerated, but also very effective with practically no side effects.

## INTRODUCTION

Idiopathic detrusor overactivity (IDO) is defined to be an urodynamic observation characterized by involuntary detrusor contraction during the filling phase.[1] International Continence Society (ICS) in 2002 characterized IDO as urgency with or without urge incontinence, usually associated with frequency and nocturia in the absence of local pathological, neurological, and hormonal factors.[2]

The incidence of IDO is as high as 17% in USA[3] and 16% in Europe.[4] Incidence of IDO increases with age.[5] The IDO significantly lowers quality of life.[3] Patients suffer with more depression, poor quality of sleep when compared with controls.[6] It leads to financial burden on the individual and the community.[7] It is postulated that approximately 30% of IDO will have incontinence.[3] Fractures; falls, skin irritations, urinary tract infections; depression, and sleep apnoea are few other co-morbidities that IDO patients will suffer from.[8] It is only recently the magnitude of this problem has been realized by general practitioners and urologists.

Anticholinergic medication has been the primary treatment for IDO patients but is associated with severe constipation, dryness of mouth, blurred vision as a result soon the therapy is discontinued.

Botulinum toxin-A (Botox-A), a neurotoxin has recently shown to be very effective in reducing detrusor contractility as it inhibits the acetylcholine release at the neuromuscular junction by cleaving the cytosolic trans location protein synaptosomal associated membrane protein (SNAP-25), thus preventing vesicle fusion with plasma membrane.[9]

Recent data from different studies have shown the role of botulinum toxin in treatment of IDO syndrome to be very encouraging.

### Study design

Our aim was to study the safety, efficacy, tolerability, and duration of the effect of 200 units of Botox-A in treating IDO patients who have failed to respond to anticholinergic agents earlier.

This is a prospective ongoing unrandomized open label study from a single center. All patients in this study were chosen to be females only as the incidence of IDO among them is higher than males.

## MATERIALS AND METHODS

Between January 2004 to June 2007, a total number of 39 females with average age of 52 years (27-69 years) with clinical symptoms of urgency, frequency with or without urge incontinence and urodynamically proven IDO without any associated or contributing neurological, hormonal, and infective pathology were included in our study. All these study group patients had a history of taking anticholinergic agent for their symptoms but either discontinued due to adverse effects or failure to respond in relieving their symptoms. None of these patients was on anticholinergic agent for the last 6 months before the start of the study.

Patients with either stress or mixed urinary incontinence, recurrent urinary infection, diabetes mellitus, or any other neurological disorder thought to be contributing to their present symptoms were excluded from our study. Patients with history of previous use of Botox-A were also excluded. None of our patients had a previous history of any bladder surgery. None of our 39 patients were either pregnant or contemplating to become pregnant during the study period.

A written informed consent was obtained from all our patients before participating in our study.

All patients underwent a thorough clinical evaluation which included full clinical history, physical examination, and maintained a 3-day urgency/frequency/volume urinary diary record prior to start of therapy. In addition all these patients underwent conventional filling cystometry study using normal saline in which cystometric bladder VFDV, maximum cystometric bladder capacity (MCC), detrusor pressure (DP), maximum flow rate (MFR), and postvoid residual urinary volume measured by catheter drainage were recorded. Filling cystometry was conducted with a double lumen urethral catheter (8F) and abdominal pressure recorded by a rectal balloon catheter (9F). Andromeda five channel urodynamic machine available in the department was used to measure all the above urodynamic parameters by a trained registrar in the department. Patients were first asked to empty the bladder and then were catheterized to drain the bladder. Patients were asked to identify the first desire to void sensation, any urgency, strong desire to void during the filling phase. Finally, the patients were asked to void with urodynamic catheters *in situ*. Urodynamic variables were measured during cystometry, i.e., VFDV, amplitude at first over active contraction, strong desire to void, MCC, and the voided volume. The MFR and Postresidual urine volume (PRUV) were also measured at the end of the urodynamic study by catheter drainage.

Our urodynamic system was regularly calibrated every 15^th^ day by company engineers.

Urodynamic assessment for all our patients was repeated at the end of 3^rd^, 6^th^, and 9^th^ month following injection of Botox-A along with clinical symptom evaluation by maintaining a voiding diary, 3 days prior to coming for urodynamic studies during follow-up.

All patients were instructed to report any time in between follow-up period on reappearance of clinical symptoms of frequency, urgency, nocturia, and incontinence.

All 39 patients received 200 units of Botox-A dissolved in 20 mL of normal saline injected intravesically into the submucosal by raising blebs in the suburothelial space at 20 different sites @1 ml (10 units) at each site sparing the trigone, ureteric orifices, and bladder dome, using a 5 mm 23 gauge Cook's needle under regional anesthesia. At the end of injection, the needle was flushed with 1 ml of normal saline so as to ensure that all the medication prepared was delivered into the area of interest in the bladder. The use of 5 mm needle tip allowed urologists to control the depth of injection into the detrusor muscle without developing extravasation into bladder serosa or placement of toxin in lamina propria.

The urodynamic variables were analyzed using nonparametric statistics and these we quote them as median variables.

## RESULTS

There were four drop outs and only 35 patients were followed up for 1 year. A total of 30 patients (85.7%) showed marked improvements in their clinical symptoms of frequency, urgency, nocturia, and incontinence within 1 week of therapy and continued to show improvement for a median period of 7 months (6-9 months) [Table 1].

**Table 1:** Result comparing clinical features before and after injection

	Baseline	3 months	6 months	9 months
Frequency/24 h	15-16 times/day	6-7 times/day	3-4 times/day	7-8 times/day
Urgency/24 h	6-7 times/day	3-4 times/day	2-3 times/day	5-7 times/day
Incontinence episod/24 h (N-8)	4-5 times/day	2-3 times/day	0-1 times/day	2-3 times/day

Urgency disappeared in 80% of our patients, incontinence resolved in 85%. Mean decrease in the number of frequency reduced from 15 to 6 episodes (−60%). Urodynamic assessment done at the end of 3^rd^, 6^th^, 9^th^ month showed improvement in VFDV and MCC in all 30 patients for a mean period of 8 months. The MCC increased by 60%. Mean increase was from 205 to 331 ml. The mean VFDV increased from 104 to 208 ml. The PRUV increased four times from the baseline value. The reduction in detrusor contractile pressure was observed in all 30 patients with a mean reduction of 49% from the baseline value was recorded. The mean detrusor voiding pressure or contractility decreased from mean 61 cm of H_2_O to 32 cm of H_2_O [Table 2]. The mean duration of efficacy lasted for 8 months (6-9 months) and then symptoms began to reappear. None of these 35 patients showed any adverse effects nor there was any incidence of acute retention of urine in our study.

**Table 2:** Result comparing urodynamic values before and after injection

	Baseline	3 months	6 months	9 months
VFDV	104 ± Nil	210 ± Nil	240 ± Nil	208 ml
MCC	205 ml	280 ml	350 ml	290 ml
DP	61 cm H_2_O	38 cm H_2_O	32 cm H_2_O	40 cm H_2_O
MRF	13-14 ml/s	9-10 ml/s	8-10 ml/s	12-12 ml/s
PRUV	20 ± 30 ml	60 ± 65 ml	85 ± 90 ml	70 ± 75 ml

## DISCUSSION

The application of Botox-A in IDO was pioneered by Schurch *et al.*[10] In her study of 19 patients, 17 patients were completely continent at the end of 6 weeks of follow-up. The MCC of bladder increased from 296.3 to 480.5 ml and decrease in maximum detrusor voiding pressure was observed from 65.6 to 35 cm of H_2_O with no side effects. The duration of effect of botulinum toxin lasted for a mean period of 9 months.

Similarly Steinhardt *et al.*[11] in their study of Botox-A showed 100% success rate on 20 patients using 200 units of the Botulinum toxin. Radziszewski and Borkowski[12] in their study of 12 patients of refractory IDO showed marked improvement in reduction of frequency, urgency incontinence rate with mean MCC increasing from 321.2 to 408.3 ml. Similar positive and improved results were observed by Loch *t al*.[13] using 200 units of botulinum toxin in IDO and Zermann *et al.*[14]

Harper *et al.*[15] in their study of 39 patients of IDO showed significant improvement in lower urinary tract symptoms with increase of mean MCC from 174 to 580 ml and the benefit lasting for 9-12 months. Verleyen *et al.*[16] in their study of 11 children with IDO showed using 125 to 250 units of botulinum has shown marked improvement in symptom relief and reduction in detrusor contraction.

Our study of 35 female patients with refractory IDO has shown similar result with significant improvement in lower urinary tract symptoms and urodynamic parameters like increase in MCC, VFDV, PRUV, and decrease in detrusor contraction pressure. We attribute our satisfactory result to suburothelial injection technique as an abundance of suburothelial sensory nerves and acetylcholine, and adenosis biphosphate containing vesicles in nerve fiber terminals have been found in the human bladder wall suggesting the lamina propria of the bladder plays an important role in transmitting the sensation of bladder fullness in the response of the bladder to stretch. This provides a greater effect on the bladder capacity increase and bladder sensation decrease subjecting that suburothelial injection of Botox-A might affect both sensory and motor function of the bladder in patients with nonneurogenic detrusor overactivity.[17] We avoided injecting into trigone and near ureteric orifices to prevent vesico-ureteric reflux of urine. Since there is a greater density of sensory nerves in this area which would theoretically be more painful to the patient apart from leading to vesico-ureteric reflux, but Christopher and Chancellor in their study injected Botox into the trigone in pediatric patients, but none of these patients experienced clinical sign of VU reflux or pyelonephritis. Similar was the result of Karsenty *et al.*[18] On an average it takes approximately 1 week before effect of toxin start showing beneficial effect.

Absolute contra indications of the use of botulinum toxin are pregnancy and myasthenia gravis.

Its use in conjunction with aminoglycosides or gentamycin should be avoided as they potentiates neuromuscular weakness.

Recent researches and clinical studies have shown enough evidence for the beneficial effects and safety of botulinum toxin in treatment of refractory IDO. The only drawback is its repeated use after certain period of time which may lead to development of antibodies and resistance to the drug.

Detrusor injection of Botox-A provides a new therapeutic choice for patients who cannot tolerate the adverse effects of antimuscarinic drugs.

The result of our study shows that the effect of suburothelial injection is much better in blocking detrusor contractility through suburothelial sensory fibers than intradetrusor injection in IDO.

## CONCLUSION

Our study of intradetrusal injection of Botox-A in management of refractory IDO has shown to be not only safe, well tolerated, but it is also very effective with practically no adverse effects with very encouraging results. But more randomized controlled clinical trials in future will establish its potential role in clinical urological practice in management of IDO and other urological diseases.
